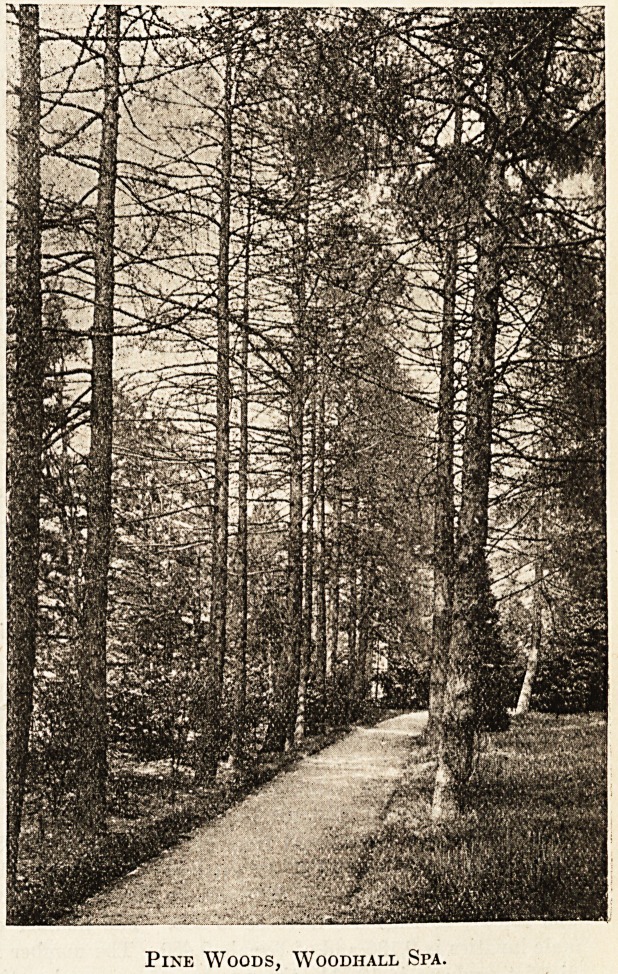# Home and Foreign Spas
*Previous articles of this series appeared in The Hospital of January 28, February 25, March 25, April 22, May 20, June 3, and June 17.


**Published:** 1911-07-08

**Authors:** 


### SPECIAL ARTICLE.

### HOME AND FOREIGN SPAS.*

### VIII.—WOODHALL SPA.

A more charming and genuine health-resort than Woodhall, or one more suited to the "rest cure," it would be difficult to find, not only in this country but throughout Europe. Unlike other spas, Woodhall is not a town, but a picturesque village of about 1,000 inhabitants, located among pine woods, between the Fens and the Lincolnshire Wolds, some sixteen miles south-east of Lincoln and twenty miles from the sea coast. Although distinctly rural in appearance, the Spa is well kept, with excellent wide roads and many pretty Queen Anne villas and cottages, whilst the scenery of the countryside is very beautiful. Owing to its quiet, restful character Woodhall Spa is an ideal spot, not for the invalid only, but also for the jaded brain-worker and those who desire to escape for a time the worries and bustle of town to find new mental vigour and bodily health amid the natural attractions of a country life, without enduring those little hardships which this so often entails.

How to Reach Woodhall.

The Spa, which is 123 miles from London, is situated on the Horncastle branch of the Great Northern Railway, and is reached by rail from King's Cross in less than three hours. The most direct route is from King's Cross *via* Peterborough, but during the season, which extends from May to October, through carriages are attached to the 4 p.m. train from the Metropolis and the 9.40 a.m. from Woodhall Spa. Special holiday and cheap week-end tickets are issued throughout the summer months at very reasonable fares.

*Previous articles of this series appeared in The Hospital of January 28, February 25, March 25, April 22, May 20, June 3, and June 17.

### The Climate and Rainfall.

Woodhall is 150 feet above sea-level, and built upon ironstone sand, through which the surface water quickly drains, so that even after heavy rain the ground rapidly dries. In consequence of the altitude, porous soil, and pine woods, the air is peculiarly bracing, dry and invigorating, and as there are no large towns or industrial centres in the immediate neighbourhood it is practically free from all contamination of smoke or fumes from factories. The average annual rainfall is 22.66 inches, which is less than that of any English spa, and the number of fine days, as well as the amount of sunshine, is proportionately great.

### The Story of the Spa.

It is due to the accidental discovery of the now famous saline bromo-iodine water that a large tract of moorland and pine woods has been, during the last seventy years, converted into one of the most picturesque and beautiful spas in Great Britain. In the year 1811 a shaft was sunk in the hope of discovering coal. When the borings had reached a depth of 520 feet a stream of water, tasting salt and of high specific gravity, rushed into the workings, and was diverted only with considerable difficulty. The boring operations were continued to a depth of 1,000 feet, but as no coal was found the enterprise was abandoned, when the deserted shaft quickly filled with water, which overflowed into a neighbouring brook. In course of time first one, then another, of the local inhabitants drank of this water, with marked relief in cases of rheumatism, gout, and other disorders, and its curative properties so impressed the then lord of the manor that in 1834 a bath house and hotel were built to accommodate visitors, who annually became more numerous as the knowledge of the healing value of the mineral water gradually spread throughout the country. For many years this constituted the Spa, and it was not until 1887 that the present development began; at this period, consequent upon the growing demand for further extension, the baths and hotel were reconstructed on a larger and more imposing scale, and every available apparatus added for the effectual employment of the mineral water in the treatment of various diseases. In 1904-05 larger adits were driven out from the main shaft of the well at a depth of 509 feet, which has resulted in nearly doubling the supply of mineral water. Every season, in fact, some addition or improvement is effected at the baths in order to keep them absolutely up to date and equipped with the very last appliance and treatment in connection with modern hydrotherapy.

The Spa Baths and Doctor's House.

### The Mineral Water.

There is no other mineral water in this country similar in character to that obtained at Woodhall Spa, and compared with the same class found on the Continent it is distinctly richer in the chlorides of sodium, calcium, and magnesium than any of the bromo-iodine waters, and quite unrivalled as regards the quantity of iodine, both free and in combination, as well as the bromine which it contains. The water percolates through a spongy stratum of rock at a depth of 512 feet, and is brought to the surface by electro-motor power in a tub holding 140 gallons at a time; as it comes from its source the water is quite clear, or of a slightly brown colour, and although possessing a marked salty flavour, it is not unpleasant to drink. It is probable that the particular layer through which the water drains was at one time the bed of a tropical salt sea, in which giant seaweeds grew, from whence are derived both the salts and the iodine and bromine compounds.

The following analysis of the water was made by Professor Wanklyn, who in his report states: "So far as I am aware this is the first instance in which free iodine has been found in appreciable quantity in a natural water":—

| | Grains per gallon. |
|---|---|
| Chloride of sodium ... ... ... | 1330.00 |
| Chloride of magnesium ... ... | 91.20 |
| Chloride of magnesium ... ... | 91.20 |
| Carbonate of soda ... ... ... | 10.00 |
| Sulphate of soda ... ... ... | .30 |
| Nitrate of soda ... ... ... ... | .55 |
| Free iodine ... ... ... ... | .20 |
| Iodine (as iodates) ... ... ... | .20 |
| Iodine (as iodides) ... ... ... | .40 |
| Bromine (as bromides) ... ... | 3.40 |
| Peroxide of iron ... ... ... | traces |

The water is used both for drinking and for baths; it is also condensed by a special process into

The Pump Room, Woodhall Spa.

the most saturated solution possible, called in German *Mütterlauge*, which is employed for special local treatments and for adding to douches and baths.

### Diseases Treated.

Taken in small quantities the mineral water produces a sedative effect upon the gastric and intestinal

Electric Bath at Woodhall Spa.

mucous membrane. In the ordinary quantities it excites the gastric secretion and helps to dissolve the viscid mucus of chronic catarrhal conditions; it also increases the functions of the pancreas and liver. When taken in full doses before breakfast it usually stimulates the peristaltic action of the bowel leading to regular emptying of the rectum. The water is indicated in, and has proved of the greatest assistance in the treatment of, muscular rheumatism, lumbago, rheumatism, osteo-arthritis, gout, catarrhal disorders of the stomach, liver derangement, neuritis, sciatica, neurasthenia, and paralysis, diseases of women, including inflammatory conditions of the uterus and appendages and uterine fibroid tumours, skin diseases, swollen glands, disorders of the throat and nose, as well as chest and heart diseases.

### The Pump Room and Baths.

The Pump Room is a spacious hall in the centre of the bathing establishment, where the mineral water is obtained by patients taking the "cure." It is warmed by radiators, lighted by electricity,

Pine Woods, Woodhall Spa.

and, being well supplied with newspapers and magazines, is the general rendezvous of the Spa.

Leading off from the Pump Room to the left and right are waiting, dressing, and cooling rooms, besides various baths, including mineral water, sulphur, pine, Aix and Vichy massage, needle and Scotch douche, and undercurrent baths. Behind the Pump Room is a two-storied building containing Nauheim, Dowsing radiant heat, electric water and electric light baths, and special rooms for throat spray, massage, electric, ionic, and $x$-ray treatments.

The baths are thoroughly equipped with the most modern apparatus, and some thirty different treatments are available, while no effort is spared to ensure that every " course " is carried out as efficiently as possible by highly trained attendants and fully-qualified masseurs. Adjoining the Pump Room is " Brookside," the charming residence of the courteous Medical Superintendent, Dr. Lionel Calthorpe, who is always pleased to afford every facility to medical men to visit the Spa and inspect the baths; and to those who may come from a distance which prevents their returning the same day to provide, free of expense, a night's hospitality at the Victoria Hotel.

A special and noteworthy feature of the Spa is the Alexandra Hospital, which was erected in 1890 at a cost of £3,000, to meet the needs of those persons to whom the expense of an ordinary course of treatment at Woodhall is more or less prohibitive. It has been twice enlarged, and now has accommodation for thirty patients, male and female. It is open to patients from all parts of the United Kingdom, admission being secured by subscriber's letter and a weekly payment of 10s. during residence, which includes baths and medical attendance. There is also the Home for Gentlewomen, the object of which is to provide at a small cost rest and medical advice to a large class of gentlewomen who otherwise would be unable to visit the Spa and take the mineral-water treatment. Ladies provided with a subscriber's letter are admitted for four weeks at 15s. per week, which includes all expenses, excepting laundry and mineral baths.

The hotels are all up to date, excellently appointed and the equal in comfort of any to be found in the Metropolis. The Victoria, which is the principal and largest establishment, stands in its own charming grounds of four acres, directly opposite the Pump Room and baths. Then there is the Royal Hotel, with its magnificent winter garden and its own well and baths, the Eagle Lodge Hotel, and the Hotel Goring, besides numerous boarding houses and apartments to suit all classes. It is possible to secure comfortable lodgings and board at a charge of from 40s. to 60s. per week, according to the position and size of rooms.

The importance of providing amusements and all forms of outdoor recreation has not been overlooked, and every effort is made to cater for the pleasure of visitors. An excellent band plays during the morning in the new band stand opposite the Pump Room and in the gardens in the afternoon, many concerts and other entertainments are given during the course of the season, and dances take place from time to time. There are excellent tennis courts and croquet lawns, and for walks and recreation the pine woods are open to visitors.

In addition there is a very fine eighteen-hole golf course, which was laid out in 1903 under the direction of Harry Vardon, and a good cricket club, where visitors are heartily welcomed, either as players or spectators. Fishing and boating can be obtained on the Witham and hunting with the Southwold, Blankney, and Burton Hounds. There are many places of interest in the district within easy distance of Woodhall Spa, including Kirkstead Abbey, Tattershall Castle, and Somersby, the birthplace of Alfred Tennyson, and as the roads are generally good and not overcrowded, motoring and cycling can be thoroughly enjoyed.

## Figures and Tables

**Figure f1:**
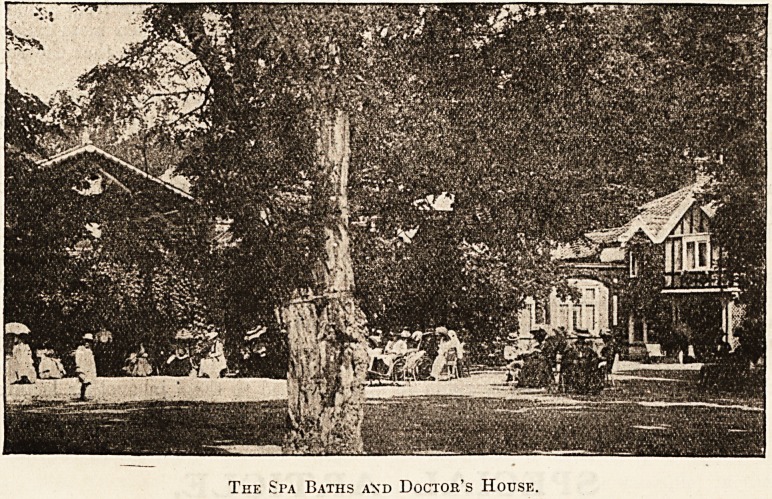


**Figure f2:**
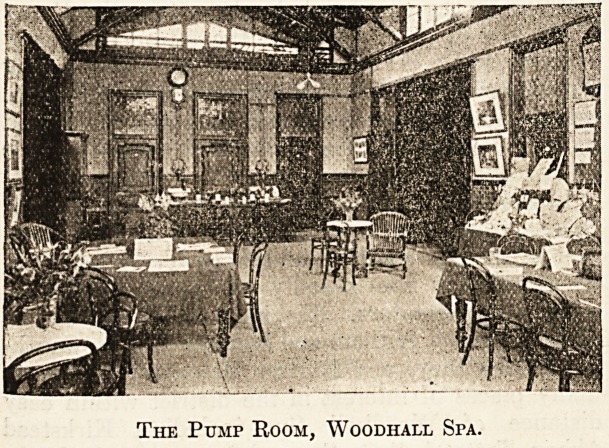


**Figure f3:**
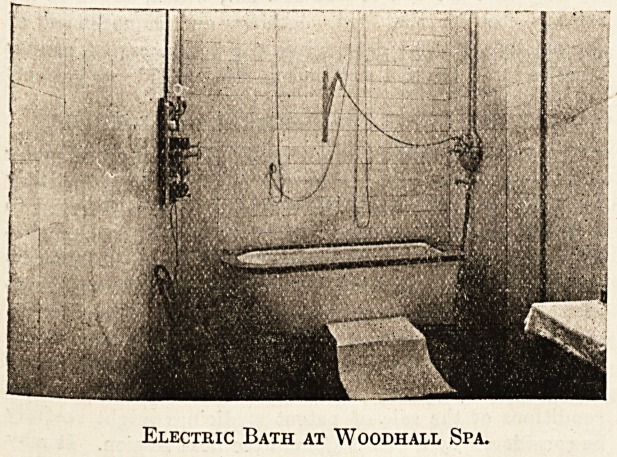


**Figure f4:**